# Neuroanatomical and Functional Correlates in Bipolar Disorder (BD): A Narrative Review

**DOI:** 10.3390/jcm14165689

**Published:** 2025-08-12

**Authors:** Anna Sara Liberati, Stefano Eleuteri, Giulio Perrotta

**Affiliations:** 1 Faculty of Psychology, Università Telematica Internazionale “Uninettuno”, 00186 Rome, Italy; annasaraliberati@gmail.com; 2 Faculty of Humanities and Social Sciences, Universitas Mercatorum, 00186 Rome, Italy; stefano.eleuteri@unimercatorum.it

**Keywords:** Bipolar Disorder (BD), neuroanatomical correlates, prefrontal cortex, anterior cingulate cortex, cerebral ventricles, amygdala, hippocampus, cerebellum, white matter

## Abstract

The incidence of mood disorders in the general population is quite high. Among the most common is bipolar disorder (BD), often associated with severe mood disorders, psychotic changes (e.g., delusions, hallucinations), impulsivity, and self-harm. However, its correct diagnosis is challenging, primarily due to the heterogeneity of clinical and symptomatic features, as well as individual differences among patients, such as comorbidity with other disorders (e.g., borderline personality disorder). Therefore, to improve understanding of its etiology and pathogenesis, refining the diagnosis must be a priority. Given the breadth and complexity of the evidence in the literature, we believe it is useful to provide a clear and comprehensive summary of the neuroanatomical and dysfunctional alterations observed, with particular attention to the prefrontal cortex, anterior cingulate cortex, cerebral ventricles, amygdala, hippocampus, cerebellum, and white matter. Through functional neuroimaging investigations it is possible to distinguish two main forms of bipolarism: the first (BD-I) is the most severe form, both in terms of manifested symptoms and in the structural and functional alterations detected; the second (BD-II) is the less severe form, which presents attenuated symptoms and mild or medium-severe alterations compared to the normotype criteria. Literature highlights the need to identify a precise study model, whether neuro-evolutionary or neuro-progressive or mixed, capable of offering clinical therapists greater scientific basis on which to anchor their diagnostic interpretations, and certainly the use of functional neuroimaging technology can be a good option even if it still presents costs that are not easily and freely sustainable by patients.

## 1. Introduction

Bipolar Disorder (BD) is a complex and pervasive psychopathology, the etiology of which involves a multitude of genetic, epigenetic, and biochemical factors that coalesce with environmental stressors giving rise to a similarly varied symptomatology. Although evidence suggests a significant genetic component, with over 64 risk loci identified [1], current genomic investigations have not yet provided definitive and comprehensive evidence of specific genetic abnormalities that could in themselves explain the etiology of (BD) [2]. This supports the hypothesis that BD may rather be a polygenic and multisystem condition that not only gives rise to physical comorbidities, such as cardiovascular disease, diabetes mellitus, immunological disorders and neuroendocrine dysfunction, but which is also linked to changes in anatomy and brain functionality. Alloy et al. [3] for example, proposed that BD may arise from a dysfunctional dopaminergic system that results in excessive reactivity in either the presence or the absence of rewards, thus explaining both the manic and depressive phases of the pathology. The authors have in fact observed, in these patients, an altered activity of the prefrontal and striatal circuits associated with the reward system, both during the obsessive search for gratification and during anhedonia.

Literature shows, in fact, numerous evidence of the existence in these patients of anomalies in glucocorticoid signaling, immuno-inflammatory imbalances, increased oxidative stress, bio-metabolic and neurotransmitter dysfunctions [4,5] inevitably correlated with variations in size and activity of various neural networks and different cortical and subcortical brain structures.

Other studies [6] have instead postulated the possible involvement of neuroinflammatory foci in the etiopathogenesis of BD, since subjects with this diagnosis often show high serum levels of proinflammatory cytokines, such as tumor necrosis factor-α (TNF-α), interleukin-1β (IL-1β) and interleukin-6 (IL-6). Considering the fundamental role of microglial cells in the release of these molecules, a particular research group [7] decided to monitor the inflammatory state of the CNS through the analysis of their activation, conducted using light emission tomography positrons (PET). In this way, a significant increase in microglial activity was detected at the hippocampus of BD patients compared to healthy controls, also directly correlating it with the presence of neuronal damage. In other works, the existence of widespread alterations in the microstructure of white and gray matter has been documented [8] together with hippocampal atrophy [9]. However, it remains to be clarified whether these alterations precede or follow the onset of the pathology.

Bipolar disorder has a strong hereditary component. Having a close relative with bipolar disorder significantly increases the risk of developing the condition. There is no single gene responsible, but rather a combination of genetic and environmental factors that interact in the development of the disorder. Studies of identical twins, who share the same DNA, show a higher concordance rate for the disorder than fraternal twins, suggesting a significant role for genetics. Several genes (Brain-derived neurotrophic factor, BDNF; Catecol-O-Metiltransferase, COMT; CACNA1C; Dopamine and GABA receptors) have been identified that may be involved in the development of bipolar disorder, including those that regulate neurotransmitters such as serotonin and dopamine, and those that influence circadian rhythms (sleep-wake cycles). However, genetic predisposition is not sufficient to cause bipolar disorder; the interaction with environmental factors, such as stress, trauma, or substance use, is also necessary, which can act as triggers [10].

Given this broad and heterogeneous clinical and symptomatic expression, research has been focusing for several years on investigating the possible neuroanatomical correlates of BD, with the aim of better clarifying its etiology and characteristics, to provide the ideal diagnostic model and functional pharmacological treatments based on the symptoms experienced.

## 2. Study Objectives

The primary objective of this narrative review is to bring together in a single publication the numerous and specific evidence currently available in the literature related to BD, for ease of consultation and understanding, in the hope that this will contribute to the advancement of knowledge about this psychiatric disorder. A secondary objective is to critically evaluate the key neuroanatomical findings and correlate them with other disorders.

## 3. Descriptive, Clinical and Diagnostic Elements of BD

BD is a chronic, progressive and serious psychiatric condition, characterized by alternating mood states oscillating between mania/hypomania, depression and mixed states, all associated with disturbances in socio-relational and sometimes cognitive functioning [11]. The incidence of the disease is rather high as it affects approximately 4% of the world population, with a certain intercultural variability regarding its expression and clinical course, while other global prevalence studies indicate an overall value around 0.5% [12,13]. BD is also very concerning from a human and social point of view, considering that it is known for being one of the psychopathologies, along with anxiety and depressive disorders, with the highest rate of suicides and attempted suicides [14] as well as the expression of behaviors at risk for one’s own and others’ safety. In fact, during episodes of mania/hypomania, when the mood is particularly elevated, the patient may experience states of extreme euphoria, hyperactivity, agitation, feelings of grandiosity, increased libido, sleep disturbances, impulsive and aggressive behavior, psychotic episodes. On the contrary, depressive phases are characterized by anhedonia, depression, melancholic mood, self-harm tendencies, vegetative symptoms and psychomotor retardation. Mixed episodes, on the other hand, are characterized by simultaneous states of depression and mania. Based on the predominance of manic, depressive or mixed tendencies, the DSM-V-TR [15] distinguishes 4 main subcategories of bipolar disorder: bipolar disorder I (BD-I), bipolar disorder II (BD-II), cyclothymic disorder or cyclothymia (cBD) and substance/medication-induced bipolar disorder.

**Figure 1:**
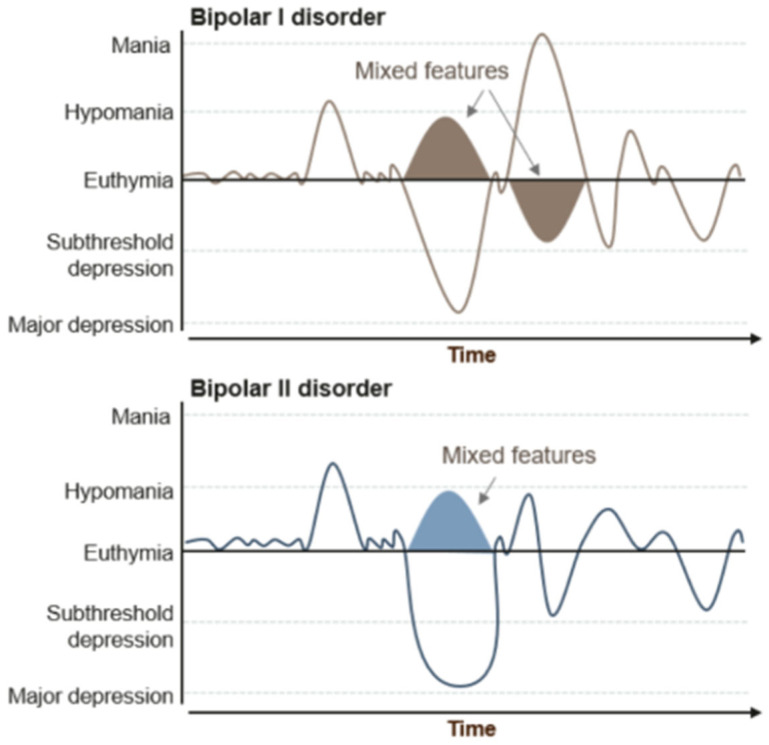
The figure shows the different alterations between the two main forms of bipolarism [20].

In details [Figure 1]:

(1) BD-I is characterized by the presence of at least one genuine manic episode, lasting at least a week, preceded or followed by hypomania and/or depressive symptoms. During the manic phases, the following can be observed: abnormally high mood, increased psychophysical energy, outbursts of anger, decreased need for rest, grandiose sense of self, logorrhea, compulsive behaviors, distractibility and lack of focused attention, disregard for risk, possibility of psychotic manifestations [16]. This mood alteration is significant enough to seriously impair the person’s social and/or occupational functioning, sometimes requiring mandatory medical treatment. For a correct diagnosis it is important to exclude that the symptoms manifested by the patient are a consequence of the use of drugs, substances of abuse or the presence of a previous medical condition. The incidence of BD-I is similar in both sexes [17].

(2) BD-II is characterized by the presence of at least one major depressive episode (recent or past) lasting at least 2 weeks, together with at least one hypomanic episode (recent or past) but not by manic episodes which, if manifested, should lead to a diagnosis of BD-I [18]. Also in this case, phases of hyper-elevation of mood alternate with depressive episodes, resulting in an overall quite uncomfortable condition for the person’s quality of life, causing clinically significant discomfort, albeit less serious than type I. For a correct diagnosis it is important to exclude that the symptoms may be a consequence of medical drugs, substances of abuse or another medical condition. The incidence of BD-II is slightly higher in women than in men [17].

(3) cBD is defined by hypomanic symptoms (e.g., euphoria, reduced sleep, excessive self-confidence) and recurrent, fluctuating, and alternating depressive symptoms, which must occur on more than half the days over a period of at least 2 years and must not be absent for more than 2 consecutive months [15]. However, since these symptoms are not sufficiently intense or of adequate duration to qualify as true hypomanic or depressive episodes, the criteria for a diagnosis of either BD or a true depressive disorder cannot be fully met.

(4) Substance/Medication-Induced BD, on the other hand, occurs during—or immediately after—exposure to substances of abuse or medications (such as cocaine or corticosteroids) or following their withdrawal. It is therefore important to underline that, although these symptoms are compatible with mania, their origin is not directly attributable to a psychopathological condition but to the effect of a chemical substance capable of exacerbating them [19].

Forms of BD associated with other medical conditions and forms of BD due to unspecified causes are also recognized. The first type is characterized by a compatible-with-mania mood disorder, although induced by a specific pathological or pharmacological condition (e.g., Cushing’s syndrome, head trauma). While BD attributed to unspecified causes can be diagnosed in the presence of evident bipolar symptoms which, however, do not fully meet the criteria for any of the other subtypes.

However, in this work we will focus exclusively on the two main types of proper BD (subtypes I and II).

## 4. The Main Neuroanatomical and Functional Correlates of BD

### 4.1. The Role of Neuroimaging Research in Bipolar Disorder

Neuroimaging-based research related to BD is often characterized by ambiguous findings and, in some circumstances, the inability to reproduce previous findings. Furthermore, several elements hinder any attempt to synthesize neuroimaging findings into a single theoretical model. The most notable of these is related to the fact that many studies fail to determine with certainty the emotional state of patients at the time of the scan, or do not provide sufficient information on their drug regimen. These factors can influence brain activity by returning data that sometimes conflict with each other. It has been noted, for example, that the effect of drugs on brain structure and function can be difficult to discern because most subjects included in the study are often treated with a combination of different drugs [21], including lithium, known to cause volumetric increases in brain regions involved in mood regulation, unlike antipsychotic and anticonvulsant drugs (for which current studies in the literature have not demonstrated the same neurotoxicity due to the lack of representative samples). Another consideration arises from the fact that most investigations were conducted almost exclusively on patients in euthymic or depressive states but less frequently on manifestly manic subjects. This should not be surprising, considering their objective difficulties in collaborating (e.g., keeping the head as still as possible during the scan) [22] but this can obviously have a negative impact on the quality and quantity of the available data. It should also be said that BD is a pathology that tends to be neuroprogressive [23] and therefore potentially characterized by different pathophysiological substrates during the various phases of its course [24]. Studies have observed that in many patients’ structural brain changes and cognitive symptoms, which are not necessarily evident from the onset of the disease, tend to worsen with the chronicity and recurrence of manic and depressive episodes. For example, some have observed that as the frequency of mood oscillations increases over time, the number of brain structures affected by volumetric reductions also increases [25,26]. Therefore, although not all individuals affected by BD manifest the same impairments, with the same severity and with the same tendency to progression, it is certainly necessary to expand the research longitudinally with the aim of identifying the factors of susceptibility to neuroprogression, and to obtain a clinical picture and a deeper understanding of the biological basis of the disorder itself.

### 4.2. Specific Areas of Interest

**Figure 2:**
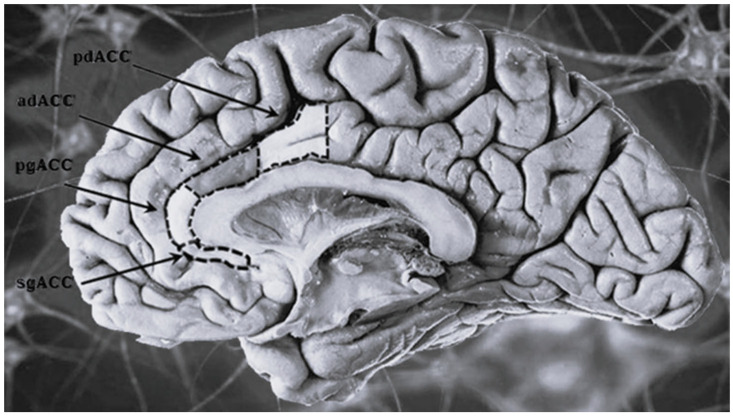
The figure shows the subregions of the anterior cingulate cortex (ACC), including the sgACC (free from copyright image, obtained from: https://debuglies.com/2020/10/26/sgacc-is-a-crucial-brain-region-links-to-depression-anxiety-heart-disease-and-treatment-sensitivity/ (accessed on 1 July 2025). original image was slightly edited with the sole purpose of better highlight the area of interest) [42].

**Figure 3:**
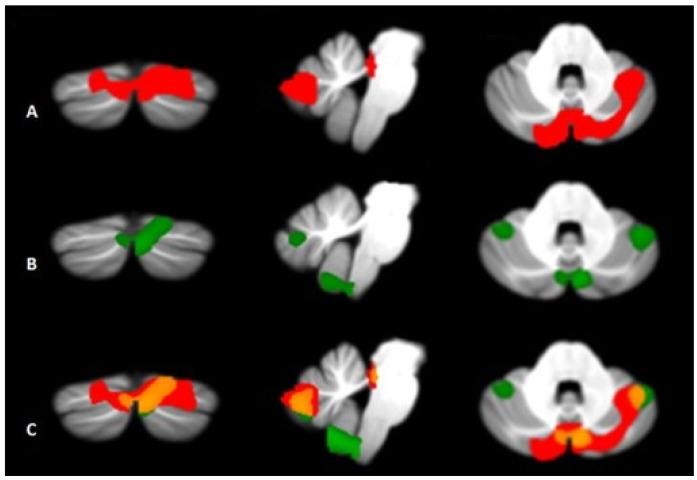
(**A**–**C**): Comparison of cerebellar density voxels of BD patients. There is a significantly reduced gray matter (GM) pattern in both BD-I (**A**) and BD-II (**B**) patients. The results are significant at *p*-values < 0.05 after correction at the FWE cluster level. The images are shown in neurological conventions. The overlapping cerebellar GM loss regions (**C**) between BD-I (red) and BD-II (green) are shown in orange (Property of: Olivito et al., 2022 [63]).

**Figure 4:**
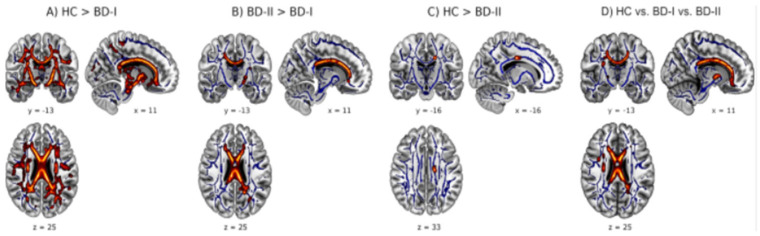
White and gray matter alterations in bipolar I and bipolar II disorder subtypes [64].

Further consideration concerns the fact that patients with BD may develop widespread and heterogeneous deficits in cognitive functions, particularly regarding emotional processing and management, inhibitory control, attention and memory skills. However, the expression and severity of these deficits differ not only according to the personal characteristics of each patient, but also in reference to the specific mood state (depressive, manic, mixed or euthymic) of the person at the time of the investigation, as well as in relation to the specific variant of BD diagnosed [27,28]. This clearly can lead to oscillations in neurocerebral activity and morphological variability, thus adding further complexity to the interpretation and replicability of the results. Nevertheless, in the literature there is fair agreement on the existence, in patients affected by BD, of some typical neuroanatomical and microstructural anomalies detectable above all in areas such as: prefrontal and anterior cingulate cortex, cerebral ventricles and various subcortical structures, in particular the amygdala and hippocampus. Noteworthy, such anomalies are compatible with those associated with other psychotic and mood disorders, such as schizophrenia, schizotypal disorder, attention deficit hyperactivity disorder (ADHD) and major depression [29,30].

(A) ***Prefrontal Cortex*** (PFC) Consistent with the presence of symptoms affecting both the cognitive and emotional spheres, PFC abnormalities are quite common in patients with BD. However, in recent years some differences have been found that seem to correlate not only with the severity and type of symptoms experienced, but also with the age of the subjects. For example, in pre-adolescent and late adolescent boys and girls, a reduction in cortical thickness has been documented particularly in the ventromedial prefrontal cortex (vmPFC) and in the ventrolateral prefrontal cortex (vlPFC), both of which are involved in emotional and behavioral regulation. These abnormalities were particularly evident in subjects with BD-I, comorbid ADHD, and those with predominantly manic symptoms [31,32]. While in peers with BD-II and in those with a prevalence of depressive states, a greater impairment of the volume and functionality of the dlPFC, a region associated with the management of functions such as working memory and sustained attention, has been documented [33] which are in fact lacking in these patients. In adults, however, anomalies have also been highlighted at the level of the uncinate fasciculus (UF), an important stretch of white matter which, running along the lower part of the lateral fissure, connects the vmPFC to the amygdala [34]. In 2018, Foley et al. [35], conducting a diffusion tensor imaging analysis, investigated the possible existence of differences in the pathophysiology of UF between subjects with BD-I and subjects with BD-II, discovering in the former the presence of a significant decrease in fractional anisotropy and greater nerve fiber impairment compared to BD-II patients, thus identifying a possible distinctive marker between the two subtypes. Finally, although studies on seniors with BD are still too scarce to draw significant conclusions, those currently present in literature [36] suggest the presence of more widespread cortical disorders affecting the prefrontal, cingulate, sensorimotor, insular, temporal, parietal and occipital, along with volumetric reductions that also affect the subcortical gray matter. The severity and extent of these abnormalities in elderly BD patients have also been correlated with a higher risk of developing neurodegenerative diseases, particularly frontotemporal dementia [37]. Although it is certainly necessary to expand the research and further investigate the nature and existence of this evidence, the data would seem to support the hypothesis of the existence of distinct pathophysiological substrates based on age and the progressive course of pathology.

(B) ***Anterior Cingulate Cortex* (ACC)**: The ACC represents an intersection between the brain’s dorsal (mainly cognitive functions) and ventral areas (mainly involved in emotional regulation), thus representing a crucial structure for the management and processing of various cognitive functions as well as for supervision behavioral responses. Several studies have reported, in subjects with BD—regardless of the subtype—the existence of a volumetric reduction of the ACC, localized in its subgenual portion (sgACC) (Figure 2) and more markedly in patients with affective and/or depressive comorbidity [38]. Since this specific subregion is involved in behavioral adaptation in relation to the relevance of emotional and motivational stimuli, its dysfunction is believed to be responsible for the inappropriate behavioral manifestations typically emitted by these patients in response to events and environmental changes perceived as unexpected, uncomfortable, or of great emotional impact [39]. An interesting piece of data comes from a study by Ongür et al. [40] in which it was demonstrated that this thinning is attributable not so much to the loss of nerve cells, but rather of glial elements. As for the comparison between younger and older patients with BD, despite the small number of studies dedicated to the latter, the data currently available in literature [41] does not seem to highlight the existence of significant age-related differences in the anomalous morphological characteristics and dysfunction of the ACC. This allows us to hazard the hypothesis that the thinning of this structure could represent a typical correlate—and therefore also a possible diagnostic marker—of BD.

(C) ***Cerebral Ventricles***: A significant meta-analysis conducted as part of the ENIGMA Project (Enhancing Neuro Imaging Genetics through Meta Analysis) the results of which were published in 2022 [43], compared data from brain scans of 307 patients with BD and 925 healthy subjects of both sexes, from over 45 different countries. The analysis found in patients, but in none of the subjects belonging to the control group, the presence of an abnormal increase—compared to the average—of ventricular volumes (progressively increasing over time), together with a thinning (also progressively worsening) of the prefrontal, fusiform, and parahippocampal cortices, the latter however mostly associated with the presence of frequent manic episodes. Although no significant differences were documented between BD-I and BD-II subtypes, it was observed that the rate of ventricular enlargement—especially the two lateral ones and the third ventricle—over time was relatively more contained in patients treated with Lithium compared to those who followed a pharmacological therapy based on anticonvulsants. These pieces of evidence would therefore seem to further support the evidence of the progressive nature of BD, as well as the hypothesis that there is an increase in the risk, for these patients, of developing a neurodegenerative pathology in old age [44]. However, as also stated by the researchers themselves, caution must be used in the interpretation of this type of data, given the still small amount of research specifically aimed at investigating the pharmacological effects on the neuroanatomical characteristics associated with BD.

(D) ***Amygdala***: Considered as the “emotional center” of the brain, the amygdala plays an important role in interpreting and managing emotionally charged stimuli, especially negative and ambiguous ones. Since many patients with BD show difficulties in interpreting the emotional meaning of facial expressions, as well as in adapting themselves to changes in environmental contingencies—to which they usually react abnormally [45]—many studies have focused on the possible role of the amygdala in BD. Although the results are not always homogeneous, in general a marked hypoactivity of this structure has been observed, especially in patients with BD-II and in those with comorbid depression [46]. This hypoactivity has been associated, rather than with the presence of specific structural anomalies, with reduced connectivity between the amygdala itself and the orbitofrontal and dorsolateral cortices [47]. In contrast, in patients with BD-I and those with a predominance of manic states the amygdala was more often hyperactive [48]. There is also ample evidence that, while the volume of the amygdala appears generally smaller than normal in younger patients, in adults it tends to increase, sometimes even exceeding that of healthy controls [49,50]. More specifically, an analysis conducted using diffusion tractography documented a direct proportional relationship between the increase in amygdala volume and the course and severity of the disease [51]. These results, therefore, seem to support the existence of a positive correlation between the progression of BD and morphostructural changes.

(E) ***Hippocampus***: There are somewhat conflicting results in the literature regarding the volume of the hippocampus in the context of BD. Some research has indeed highlighted an increase in hippocampal volume in patients compared to control subjects [52]. Others, on the contrary, have reported a reduction localized especially in the regions of the Cornu Ammonis (CA), Dentate Gyrus (DG), and subiculum, more significant in patients with BD-I [25], while others have not found significant differences between bipolar patients and the normal population [53]. There are several pieces of evidence that report larger hippocampal sizes in younger BD patients compared to adults, although correlations have been observed between hippocampal hypertrophy and therapy based on mood-stabilizing drugs (such as Lithium), which are believed to have an anti-atrophy effect [54]. On the contrary, in adult patients, regardless of whether or not they use drugs, a progressive decrease in hippocampal density has been documented and, more generally, in parahippocampal cortical structures which are hypothesized to be associated with the presence of a genetic polymorphism involved in the regulation of BDNF functionality (val66met) known to play a role in the neuroanatomical and cognitive anomalies commonly observed in patients with BD [55,56].

(F) ***Cerebellum***: although the cerebellum is typically associated with predominant motor functions (e.g., coordination and learning), this important structure is also involved in the management of cognitive and emotional functions, including mood regulation and social behavior [57]. This is thanks to the numerous connections it establishes with areas such as the PFC, the basal ganglia, and the limbic system. Since several studies conducted both on patients with cerebellar lesions [58] and on people suffering from psychopatho- logies [59] have highlighted alterations in the emotional and behavioral sphere, it has been hypothesized that the cerebellum may also play some role in the etiology of symptoms associated with BD. Functional imaging studies have highlighted a pattern of significant atrophy affecting various regions of the cerebellum in bipolar patients. These regions include the vermis, anterior lobe V, and posterior lobules Crus I and II [60,61], which are known for their close connection with frontal, temporal, and limbic areas. Furthermore, a recent fMRI investigation conducted on subjects at rest [62], in addition to demonstrating that alterations in functional connectivity between the brain and cerebellum persist even during the euthymic phase observed some distinctive characteristics between BD-I and BD-II subtypes. In the former, an alternating pattern of hyper- and hypo-cerebellar-cortical connectivity was found, while BD-II patients showed exclusively hyper-connectivity patterns. To compare the gray matter density of the cerebellum between the two subtypes, the same research group also carried out voxel-based morphometric analyses. Despite the presence of some common anomalies (Figure 3C), in patients with subtype I, a reduction of the anterior and posterior cerebellar portions was observed, more evident on the right (Figure 3A), while BD-II subjects showed a pattern of diffuse atrophy bilaterally (Figure 3B). According to the authors, these differences could reflect the different clinical characteristics that denote the two subtypes. However, there are currently no known specific studies aimed at investigating possible cerebellar changes in BD patients distinguished based on age and progression of the disorder, therefore this could represent an interesting hypothesis for future research.

(G) ***White and gray matter***: In bipolar disorder, alterations in the brain’s white and gray matter have been associated with changes in the connectivity and volume of these areas, particularly those involved in emotional and cognitive regulation (Figure 4). These changes may contribute to the mood variability and cognitive deficits observed in bipolar patients. White matter, composed primarily of myelinated nerve fibers, is critical for communication between different brain areas. Neuroimaging studies have revealed reductions in white matter volume and alterations in its structural integrity in individuals with bipolar disorder. These alterations may affect the speed and efficiency of nerve signal transmission, potentially impacting mood regulation and cognitive function. Gray matter, composed primarily of neuronal cell bodies, is involved in cognitive, emotional, and behavioral functions. Researchers have observed reductions in gray matter volume in several brain regions in bipolar patients, such as the frontal, temporal, and limbic lobes, which are important for emotional control, social cognition, and memory. These reductions may contribute to the affective and cognitive symptoms of bipolar disorder, such as difficulty regulating emotions, memory problems, and impaired thinking. Alterations in white and gray matter in bipolar disorder suggest a neurobiological basis for the disease. These changes may be related to the disorder’s specific symptoms, such as mood swings, cognitive deficits, and difficulties with emotional regulation. Understanding these alterations may lead to new therapeutic strategies to address bipolar disorder symptoms and improve patients’ quality of life [64].

In literature, the age factor is relevant. Although published studies present various selection and representativeness biases [65], age is an indicator of complexity and symptomatic differentiation among patients. The most impactful studies distinguish between four main populations: children, adolescents, adults, and the elderly. The differences observed concern the severity of symptoms and the negative impact on structures and functioning, demonstrating that, regardless of genetic pathologies that determine its own severity, bipolar disorder is correlated with age. Studies with larger populations are desirable to more clearly distinguish the differences between the categories of bipolar disorder.

Literature highlights the need for mental health, neurology, and psychology professionals to identify a specific study model. Literature currently offers three main approaches: one oriented toward neuro-developmental and neuro-progressive studies, using functional neuroimaging technology, or a mixed model, which can provide clinical therapists with a stronger scientific basis for their diagnostic interpretations based on the patient’s symptoms. Conversely, however, there is the alarming fact that the use of functional neuroimaging technology, despite being a valuable investigative option, still presents costs that are not easily and freely affordable for patients, as they are either entirely borne by the patients themselves or through funded studies and research, with relatively lengthy execution and analysis times [33,36,66].

Below is a summary table of the neuroanatomical differences between a subject with BD and a healthy one (Table 1).

Below is a summary table of the neuroanatomical differences between a subject with BD-I and BD-II (Table 2).

## 5. Clinical Implications

A correct diagnosis of bipolar disorder is crucial to starting effective treatment and improving the patient’s quality of life. A misdiagnosis, often as major depression, can lead to ineffective and, in some cases, even harmful treatment, with the risk of worsening the course of the illness.

Importance of accurate diagnosis: Targeted treatment: A correct diagnosis allows for the most appropriate treatment, which may include mood-stabilizing medications, psychotherapy, and, in some cases, additional therapies. Early intervention with appropriate treatment helps reduce the risk of relapse, both in manic and depressive phases, and stabilize the patient’s mood. Reduction of suicide risk: Bipolar disorder is associated with a high risk of suicide, especially during depressive episodes. Early diagnosis and timely treatment can reduce this risk. Timely diagnosis and treatment can help prevent the functional deterioration that can occur due to repeated episodes of mania or depression.

Bipolar disorder can also be associated with other medical and psychiatric conditions; therefore, an accurate diagnosis helps identify and treat these conditions as well, further improving the prognosis. Finally, in the literature, there are several predictors to be taken into consideration to systematically distinguish between the two main forms of bipolarism, as shown in Table 3 [66].

Therefore, a correct diagnosis can shorten the duration of untreated illness, which can negatively impact the patient’s prognosis and quality of life.

## 6. Study Limitations

This narrative review only minimally addresses limitations related to contradictions or methodology. These include the difference between cross-sectional and longitudinal imaging, drug effects, and sample heterogeneity. This choice is because the purpose of this review is to identify neurostructural and functional alterations in a single study, before proceeding with a meta-analysis of the literature in a future publication.

For this reason, we acknowledge that the findings presented in the literature are also conflicting and cannot provide sufficient explanations for the etiological nature and neuroanatomical correlations, even in relation to other related disorders such as depression, mania, and bipolar disorder, which all present the same alterations, albeit with significant differences due to hyper- or hypo volumetry.

Future studies should take these critical issues into account, using representative population samples, guiding research using longitudinal neuroimaging, to measure structural and functional changes, from the first symptoms (even if mild) and connectivity-based subtyping, with reference to the identification of pathological subgroups using abnormalities in brain connectivity as investigative markers.

## 7. Conclusions

Despite the vast amount of data and evidence in literature, a clear and unique etiopathogenetic model capable of accounting for the extreme heterogeneity of symptomatic manifestations and brain alterations associated with BD and its evolution over time is not yet available. The clinical and physiological manifestations of BD are complex and varied, and the sudden and constant oscillations of mood represent only the most evident sign. Therefore, although several risk factors, mainly of a genetic and environmental nature, have been identified in recent years, the neurophysiopathology of this condition remains, to date, still unclear.

One limit to this understanding is given by the fact that most studies are still mostly cross-sectional, and therefore undoubtedly able to identify associations, but not cause-effect relationships between the variables involved, nor to differentiate the changes associable with the course of the disease itself among clusters of patients in different developmental stages and affected by one or the other subtype of the disorder itself. This partly could explain the presence of contrasting research data and evidence regarding the characteristics of the neuroanatomical correlates associated with bipolarism.

A second limit is linked to the fact that BD involves heterogeneous symptomatology as well as a wide range of co-causal factors of both genetic and environmental types. However, due to the absence of specific biomarkers, the diagnosis is still mainly conducted based on purely clinical criteria, which include interviews and behavioral observations of the patient.

Therefore, since BD overlaps physiopathologically with several other disorders—often present in comorbidity—its correct identification is often subject to evaluation errors that not only slow down the correct therapeutic and pharmacological intervention but can contribute to increasing the stress and frustration of the patient, risking exacerbating the symptomatic picture even more.

In fact, in addition to the complexity itself that characterizes this psychopathology, the neuro-cerebral functionality of the patients who are affected by it is constantly influenced also by exposure to contingencies and environmental stressors, which can further contribute to the neuroanatomical changes associated with BD and its subtypes, further complicating the possibility of discriminating the predisposing factors from the factors consequent to the development of the disorder itself.

Hence the need to adopt a more holistic research approach, which considers not only genetic characteristics, but also the complex interactions that occur between the genes themselves, the environment and the person’s neuro-cerebral and physiological development. In this regard, a possible research direction could include not only longitudinal observations, but also the adoption of an experimental model based on endophenotypes.

Considering that endophenotypes allow us to represent more elementary phenomena compared to the very complex behavioral ones on which the diagnostic approach is currently based, their identification could facilitate the creation of etiopathogenetic models of BD and its subtypes—also considering its progressive nature—which can be useful both at therapeutic and at a diagnostic level.

Expectations for future studies are oriented towards a multifactorial analysis of the neuropathological phenomenon, using more representative population samples, guiding research using longitudinal neuroimaging and connectivity-based subtyping.

## Figures and Tables

**Table 1:** Neuroanatomical and functional differences between a healthy subject and a subject with BD.

Neuroanatomical Areas	Healthy Subject (Average Adult)	Person with BD
*Prefrontal Cortex* *(PFC)*	Located anteriorly to the frontal lobe, the PFC—one of the last regions of the cortex (neocortex) to develop—plays a pivotal role in executive function, orchestrating, together with other areas with which it is reciprocally connected, a complex symphony of motor, cognitive and emotional functions. Specifically, vmPFC and vlPFC are involved in emotional and behavioral regulation, while dlPFC is associated with cognitive functions such as working memory and sustained attention.	In adolescents of both sexes, a reduction in cortical thickness has been documented particularly in the vmPFC and vlPFC. These abnormalities seem particularly evident in subjects with BD-I, comorbid ADHD and those with predominantly manic symptoms. In peers with BD-II and those with a prevalence of depressive states, an impairment of the volume and functionality of the dlPFC has been documented. In adults there are also anomalies at the level of the uncinate fasciculus (UF), more serious in patients with BD-I. In seniors abnormalities in PFC are widespread, thus leading to an higher risk of neurodegenerative disorders. Functional MRI studies have shown increased activity in the ventral striatum and left prefrontal cortex during reward processing tasks in patients with bipolar disorder.
*Amygdala*	Placed bilaterally in the anterior portion of each of the medial temporal lobes. Reaches an average vol. of ≈2.30 ± 10 cm^3^ (larger on the right), wider in males. It consists of 13 distinct nuclei, each one with its own functions and connections to other brain structures. Overall, it participates in emotional and olfactory memories, sensory input processing, emotion managing—particularly anger and fear- and their behavioral, neurovegetative and hormonal responses.	Although the results are not always homogeneous, a marked hypoactivity of this structure has been observed especially in patients with BD-II and in those with comorbid depression. In contrast, in patients with BD-I and those with a predominance of manic states the amygdala is generally hyperactive. Also, while the volume of the amygdala appears generally smaller than normal in younger patients, in adults it tends to increase.
*Hippocampus*	Bilateral medial temporal lobe fold of mean length ≈ 8 cm. Diffusely innervated by afferent and efferent fibers to other CNS structures. Principal Center for Memory and Learning, also handles functions of spatial orientation, intra- and extractor-portal sensory and perceptual processing, object recognition, socioemotional info processing and subsequent behavioral responses, and stress management.	Although scientific findings are quite inhomogeneous, a large amount of data show a larger hippocampal volumes in younger BD patients compared to adults. On the contrary, in adult patients a progressive decrease in hippocampal and parahippocampal density has been reported, probably associated with the presence of a genetic polymorphism involved in the regulation of BDNF functionality (val66met).
*Cerebral Ventricles*	There are four cerebral ventricles: two lateral ones are situated within each hemisphere of the cerebrum. The third ventricle is located in the diencephalon, between the right and left thalamus, while the fourth is situated at the back of the pons and upper half of the medulla oblongata. The ventricles produce and store cerebrospinal fluid (CSF, approximately 20–25 mL in total), which surrounds the brain and spinal cord, providing protection from trauma. CSF also removes waste and delivers nutrients to the brain. The choroid plexuses in each ventricle are responsible for the synthesis of CSF itself.	Patients with BD often show an abnormal and progressive increase of ventricular volumes, together with a thinning (also progressively worsening) of the prefrontal, fusiform, and parahippocampal cortices, the latter however mostly associated with the presence of frequent manic episodes. No significant differences were documented between BD-I and BD-II subtypes.
*Anterior cingulate cortex* *(ACC)*	The most distal portion of the cingulate gyrus (bilateral structure surrounding the corpus callosum). Given the direct connections it establishes with the prefrontal cortex and some limbic structures (amygdala, hypothalamus, and hippocampus) it participates in the encoding of emotions particularly anxiety, anger and fear. Also it regulates some endocrine and vegetative functions and participates in emotional language production.	Regardless of the subtype it has been documented the existence of a general volumetric reduction of the ACC, localized in particular in its subgenual portion (sgACC), more evident in patients with affective and/or depressive comorbidity. No significant age- or subtype- related differences have been reported between patients.
*Cerebellum*	Located in the posterior cranial fossa behind the pons and medulla oblongata, separated from them by the fourth ventricle. It is divided into two hemispheres (left and right) and three lobes (anterior, posterior and flocculonodular). It is responsible for dealing with motor learning, coordination and precision of motor functions, but it also plays a role in cognitive, emotional, linguistic and visuospatial functions, thanks to connections [cortico-ponto-cerebellar (CPC) and the circuit cerebello-thalamo-cortical (CTC)] with the frontal, temporal, parietal cortices and paralimbic regions.	Functional imaging studies have highlighted a pattern of significant atrophy affecting various regions of the cerebellum (including the vermis, anterior lobe V, and posterior lobules Crus I and II), with some distinctive characteristics between subtypes: in BD-I it has been observed a reduction of the anterior and posterior cerebellar portions, more evident on the right hemisphere, while BD-II subjects show a pattern of diffuse and bilateral cerebellar atrophy.
*White matter*	White matter is a component of the central nervous system, consisting primarily of myelinated nerve fibers that connect different areas of the brain and spinal cord. Myelin, a fatty substance that coats axons, gives them their characteristic white color and allows for the rapid transmission of nerve impulses. White matter is essential for communication and information processing within the nervous system.	An increase in deep white matter hyperintensity (areas that show abnormal signal intensity on MRI) is also observed.
*Grey matter*	Gray matter consists of the cell bodies of neurons and is primarily found in the cerebral cortex.	Reductions in gray matter volume may be observed in specific brain areas, such as those involved in emotional control and reasoning. These reductions may be associated with difficulties regulating emotions and making decisions.
*Cortical thickness*	Cerebral cortical thickness, which refers to the thickness of the cerebral cortex, is generally between 2 and 4 mm. The cortex, a layer of gray matter, is the outer surface of the brain and is rich in neurons. Its thickness varies slightly depending on the different areas of the brain, but generally remains thin, about 2–4 mm in adults.	Although there are no significant changes in cortical surface area, reduced cortical thickness has been reported in areas such as the operculum and midfrontal cortex.

**Table 2:** Neuroanatomical differences between BD-I and BD-II. Source: Authors.

Neuroanatomical Areas	Person with BD-I	Person with BD-II
*In general*	The neuroanatomical differences between bipolar disorder type 1 and type 2, although not yet fully understood, primarily concern the extent and severity of mood episodes. Type 1 is characterized by full-blown manic episodes, potentially with psychotic symptoms, while type 2 presents hypomanic episodes (less severe than mania) and major depressive episodes. This is reflected in some neuroanatomical differences, with type 1 showing greater impairment in certain brain areas involved in emotional processing and mood regulation. In DB-1, the presence of manic episodes with possible psychosis and greater functional impairment suggest greater neuroanatomical dysregulation, involving larger brain areas. In DB-2, with the predominance of hypomanic and depressive episodes, it may have a less marked impact on some brain areas, although the specific differences are not yet fully elucidated.	
*Hippocampus*	The volume is markedly reduced, generating a greater impairment of functions such as memory, learning, problem solving and mood regulation.	The volume is reduced but significantly less than the DB-1.
*Prefrontal cortex*	The volume appears significantly reduced, generating a greater impairment of functions such as planning and problem solving, with a greater tendency towards impulsivity.	The volume is reduced but significantly less than the DB-1.
*Anterior cingulate*	The volume appears moderately reduced, generating a greater impairment of functions such as emotional processing and the management of fear and frustration.	The volume is reduced but significantly less than the DB-1.
*Cerebral ventricles*	The volume appears significantly increased, with greater presence of fluid in the ventricular spaces, generating a greater impairment of functions such as memory, attention, executive function and planning, favoring degenerative dementia processes.	Volume increased but significantly less than DB-1.
*Cerebellum*	Reduction of the anterior and posterior cerebellar portions, with greater evidence in the right hemisphere.	Diffuse and bilateral cerebellar atrophy.
*Grey and White matters*	Significant structural and functional reduction	Mild or moderate structural and functional reduction

**Table 3:** The table shows the different alterations between the two main forms of bipolarism [66].

Different Alterations Between the Two Main Forms of Bipolarism (BD-I and BD-II)		
BD-I	Elements Shared in Both Forms but with Different Intensity and Frequency	BD-II
Anxiety disorders and Emotional reactivity	Cyclic nature of manic and depressive symptoms	Age of BD onset
Metabolic syndrome	Residual mood symptoms	Number of episodes
Poor patient cooperation and adherence to therapy	Poor Sleep quality	Patient cooperation and adherence to the- rapy
Psychiatric drugs such as antidepressants, mood stabilizers, and antipsychotics	Childhood trauma	Psychiatric drugs such as antidepressants and mood stabilizers

## Data Availability

Not applicable as no new data is generated in this review article.
